# Land-based foraging by polar bears reveals sexual conflict outside mating season

**DOI:** 10.1038/s41598-024-71258-w

**Published:** 2024-08-31

**Authors:** Jouke Prop, Jeffrey M. Black, Jon Aars, Thomas Oudman, Eva Wolters, Børge Moe

**Affiliations:** 1 Arctic Centre, University of Groningen, Groningen, The Netherlands; 2 Department of Wildlife, Cal Poly Humboldt (formerly Humboldt State University), Arcata, CA USA; 3 FRAM Centre, Norwegian Polar Institute, Tromsø, Norway; 4 Branta Research, Ezinge, Groningen, The Netherlands; 5 Norwegian Institute for Nature Research (NINA), Trondheim, Norway

**Keywords:** Intrasexual selection, Familiarise, Forager, Male competition, Solitary carnivore, Ecology, Evolution, Zoology

## Abstract

According to sexual selection theory, the sexes are faced with opposing evolutionary goals. Male fitness benefits from access to females, whereas female fitness is constrained by food resources and safety for themselves and their offspring. Particularly in large solitary carnivores, such as polar bears (*Ursus maritimus*), these divergent goals can potentially lead to conflict between the sexes. Outside the mating season, when polar bears are on the move across vast distances, the consequences of such conflict can become apparent when individuals arrive at the same food source. To investigate interrelationships between the sexes, we observed successive polar bears visiting a bird breeding colony to feed on clutches of eggs. We found that males succeeded females more frequently and more closely than expected by chance. Moreover, when males were closer to conspecifics, they walked faster, spent less time in the colony and ingested less food. In contrast, female foraging performance was not associated with proximity to other bears. Irrespective of proximity, females generally spent short periods in the colony and ingested fewer clutches than males. Our results suggest that in polar bears, there is a trade-off between the benefits of food intake and the opportunities (in males) and risks (in females) posed by encountering conspecifics.

## Introduction

Within the framework of sexual selection theory, males and females are expected to differ in how they resolve the trade-off between approaching and avoiding others, resulting in a wealth of mating systems across the animal kingdom^1,2^. For example, in solitary carnivores, where females raise offspring alone, the fitness of females may be constrained by day-to-day safety for themselves and accompanying offspring^3,4^ due to risks of sexual harassment or infanticide from larger males^4–6^. In contrast, the fitness of males may be constrained by access to receptive females^7^, which may depend on opportunities to locate potential mates as well as potential competitors well before the breeding period^8,9^. Such conditions may lead to the emergence of conflict between the sexes^10,11^ with respect to the proximity that males and females prefer to maintain on the landscape, linked to future reproductive success in males and safety (or survival) in females. Solutions to daily challenges arising from this conflict may include monitoring the proximity of conspecifics and adjusting foraging decisions and movements in order to either get closer, in the case of males, or maintain safe distances, in the case of females^12–14^.

From an optimal foraging theory perspective^15^, foraging decisions must balance the benefits of nutritional gains and costs of finding food, including avoiding predators and competitors^16,17^. In male carnivores, costs may arise due to the incompatibility of foraging and other competing activities that determine fitness^18,19^, such as finding mates. Choosing to forage will lead to costs associated with spending less time investing in the other activities^20^, and the severity of these costs may depend on distance to conspecifics. Close proximities imply a larger previous investment of time and energy in getting close as well as a larger potential loss of proximity benefits if contact is not made^20^. In female carnivores, lengthy sittings with a prey incur increased probability of encountering males that are attracted to the same prey^21,22^ with mortality risks to the female or associated offspring^23^. These costs may be reduced by alternating between high-risk foraging habitat and low-risk refuges, where food is digested during resting periods^16,24^. Thus, we infer from optimal foraging theory that in solitary carnivores, proximity to conspecifics has an impact on foraging activities. Although such relationships may be universal, actual field observations are sparse because observations on foraging behaviour may be lacking^25,26^ or spatial positions relative to conspecifics may be unknown^27^. Yet, the interplay between foraging performance^28,29^ and proximity adjustment is key to appreciating the ecological impacts of such potential conflict^30^.

Polar bears (*Ursus maritimus*) are solitary carnivores in which full-grown males have twice the body mass of females^31^. Both sexes have finely tuned adaptations for hunting seals on Arctic sea ice^32,33^, with the most important species for polar bears being ringed seals (*Pusa hispida*)^34^. Males as well as females exhibit seasonal site fidelity, although the strength may vary among populations^35,36^. Polar bears are non-territorial and their mating system falls within the category of “roving males”^7^. This means that males and females are distributed at low densities, and only consort during the mating season (March–May) when males actively search for females that signal receptiveness to mating through the scent left in their tracks, urine marks, and scat^34,37^. For example, Laidre et al.^38^ monitored the travel paths of male and female polar bears marked with satellite transmitters. Using information on the angle between subsequent steps of individual bears and distances between subsequent locations across days and weeks, Laidre et al.^38^ calculated the paths of females to be more linear and those of males more tortuous during the breeding season. With the aid of simulations, Laidre et al.^38^ hypothesised that this disparity can be explained if males are assumed to follow females while also avoiding encounters with other males.

The arrival of spring in the Arctic signals the start of the brief breeding season. Given the strong competition among males for access to females^39^, we propose that male polar bears may prepare for successful breeding not only during this period but also throughout the year by getting closer to females. The selective advantages of periodically “closing the proximity gap” might include opportunities to investigate scent marks, which may communicate information on females’ qualities. Males may also advertise personal qualities to females to smoothen interactions when they meet later during the mating season^40^. Furthermore, male polar bears might gain a direct reproductive benefit from following females, as polar bears occasionally mate after the usual breeding season as late as June or July^41,42^. This may occur after a cub dies and the female returns to estrus^41,43^. In contrast, female polar bears may be selected to avoid males outside the breeding season, and several polar bear populations exhibit strict sexual segregation^44–46^. In populations where polar bear sexes are not segregated, infanticide and femicide by males occur^5,47,48^.

Due to climate change, the sea ice in many parts of the Arctic does not fully freeze in winter and quickly thaws in spring. This leaves limited opportunities for land-based bears to reach retreating pack-ice^49^ and the seals that form their staple food. Concurrently, polar bears have increasingly been observed to exploit terrestrial food sources, including berries, fish, reindeer (*Rangifer tarandus platyrhynchus*), birds and bird eggs^50–55^. In Svalbard, polar bears have discovered an alternative food supply on offshore islands and cliffs where birds establish nesting colonies^50,51,56^. During several years, polar bears were found to eat more than 90% of the eggs produced by gulls, ducks, and geese in a single colony^56^. The birds’ annual return to the colonies offers a window of opportunity for foraging polar bears each summer from the beginning of June until mid-July^56^. During this annual window, these bird colonies also allow for unique opportunities to observe the arrival, departure, and foraging performance of individual polar bears.

If a “closing the proximity gap” phenomenon operates on the Svalbard coastline for polar bears, it should be possible to distinguish sex-mediated travel patterns. These include adjustments in the speed or pace of continuing along travel paths depending on “who” or at least which sex has passed through an area. The sex of the previous bear may influence how quickly the next bear proceeds because of a trade-off between time spent foraging in the bird colony and adjustments in proximity. In order to test these predictions and explore evidence for conflicting adaptive strategies in males and females, we determined the positions of polar bear individuals relative to others, and linked these positions to foraging behaviour in the bird colony. First, if males follow females, this would become apparent in the order that bears visit the bird colony and time spans between successive bears. Thus, we predict males to more often succeed females and at shorter intervals than expected by chance alone. Further, bears are expected to adjust the time spent foraging and resting in the colony with respect to the distance of other polar bears. More precisely, we predict males to effectively close the gap in distance to another bear, and to females in particular, and to spend less time in the colony, take fewer clutches, and consequently be less likely to take rest breaks when other bears are proximate. If female polar bears are followed by males, they are restricted in how to sense their followers. Namely, a bear that follows the travel path of another bear likely uses olfactory cues from tracks, scat, and urine left on the landscape to gain information^57,58^, whereas those being followed will have less information about their followers unless scent plumes are within range and local weather conditions are favourable^59^. Female polar bears, therefore, may not rely on proximate cues as the prime source to detect and locate others. Rather, in line with abundant empirical work on abilities of foragers to cope with the “average” predation risks in their environment^20,60^, female polar bears are anticipated to exhibit more moderate responses while foraging in the colony. That is, since the bird colony can be viewed as a high-risk habitat for females, we expect females to reduce taking rest breaks in the bird colony, to stay for shorter times in the bird colony than males, and take fewer clutches. If true, this would indicate that foraging decisions by polar bears are constrained by the way the sexes interact, which may have consequences for nutritional gain.

## Methods

### Study area and study setup

**Fig. 1:**
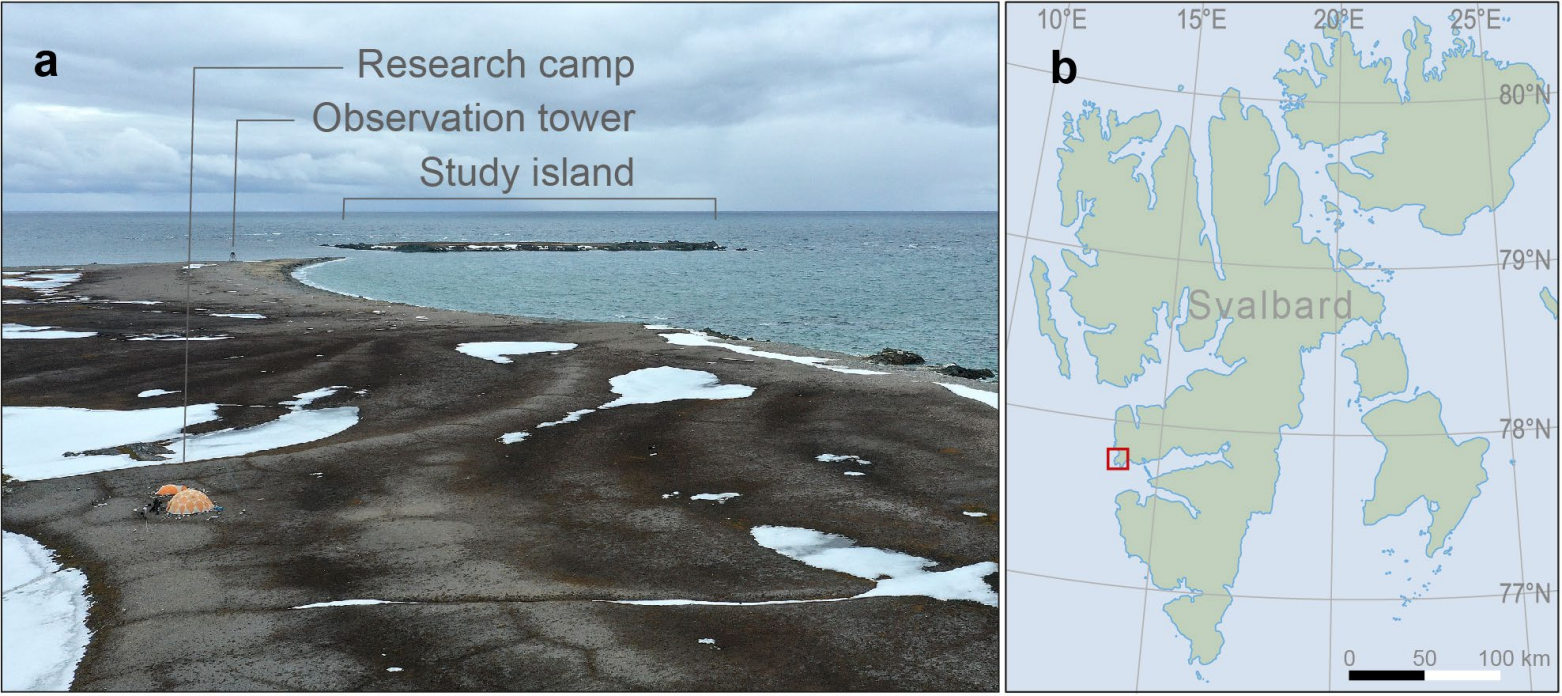
Overview of part of the study area (**a**), with the study island in the background, observation tower on the beach wall, and research camp in the foreground. The study area (**b**) is located on the west coast of Svalbard, Norway, (within the red square).

The study area is located on Nordenskiöldkysten at the west coast of Spitsbergen, the largest island of the Svalbard archipelago (Fig. 1). During the winter, polar bears reside in the adjacent fjord complex of the Van Mijen–fjorden–Bellsund area^31,61^. In this fjord complex, mating takes place during the months of March to May^62,63^. Polar bears used to roam the sea ice expanses around Svalbard^62^ but with decreasing sea ice, an increasing number of polar bears spend much of the summer on land^49,64^. With retreating sea ice in spring, polar bears initiate movements passing through Nordenskiöldkysten, usually within 1 km of the coastline, with detours inland. The polar bear population under study, which resides on land during summers, does not exhibit sexual spatial segregation, and males and females use the same stretches of land. The landscape is generally open with a variety in structure, such as beach ridges, river beds, inland raised beaches, and mounds of up to 55 m in elevation. Snow beds may persist until early July, but otherwise the study area is not covered by snow from mid-June onwards. Along the coast, greenstone islands are home to breeding barnacle geese (*Branta leucopsis*), common eiders (*Somateria mollissima*) and glaucous gulls (*Larus hyperboreus*). One of these islands is Diabasøya (77.77°N 13.71°E), which hosts the main study colony of birds and is 3 ha in size and 150 m offshore^65^. A 6-m tall tower on the beach wall opposite the island provides views over the island and surroundings. The tower was used for observation for most of the time that birds occupied nests on the island, which lasts from the end of May through mid-July^56^. During the time that the tower was not used, observations were continued from a research camp that was located at 800 m from the island. To check for the unlikely event that a polar bear visit had been overlooked, we operated Bushnell wildlife cameras from the tower to take photographs once per minute. From the tower and camp, polar bears could be spotted at distances of up to 8 km as long as atmospheric conditions allowed, which gave the opportunity to prepare an observation session as soon as a polar bear approached the study area. In this study we focus on observations in the core study area within a radius of 2 km of the observation tower (hereafter “study area”). Data were collected in 2009–2022 from the end of May through end of July. The field seasons of 2020 and 2021 were exceptions in that they started six weeks later, and these years were therefore not used for calculating seasonal bear presence.

To avoid disturbing polar bears and breeding birds, the colony island was not visited during the birds’ incubation period. Instead, nests were mapped from the observation tower to determine nest availability for each observation day. The locations of bird nests (barnacle goose, eider and glaucous gull) were marked on high-resolution photographs of the island and updated daily throughout the breeding season to give the number of nests mapped (*NAM*_*ds*_) for species (*s*) and date (*d*). Not all nests were visible, such as when the view was obstructed by rocks. Visibility of nests was determined by an inventory of the island after the breeding season, which gave the proportion of visible nests (*PV*) averaging at 0.62 (± 0.041 SD, n = 12 years). We assumed that the visibility of nests remained constant throughout the season, giving the daily number of nests available as *NAM*_*ds*_/*PV*. Food availability was expressed as densities (number of nests per ha).

### Polar bear observations

**Fig. 2:**
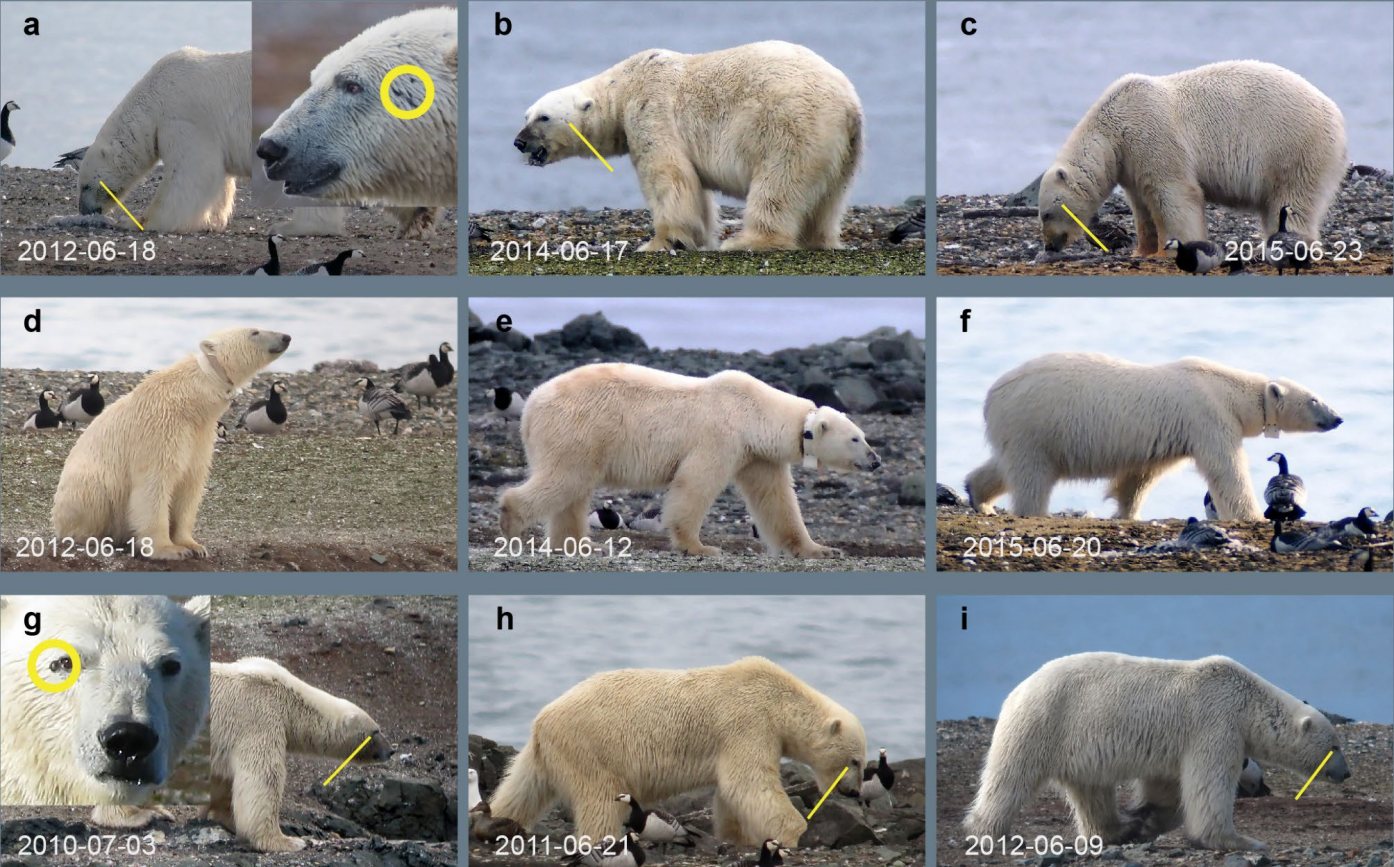
Identification of individual polar bears combines assessing the build and shape of the bear as well as exploring finer details. Example sets show three individuals observed at different occasions: a male with scar on its head (**a**–**c**), a female recognisable from head and body shape, and GPS collar (**d**–**f**), and a male with a tiny protuberance just beside its right eye (**g**–**i**). Dates of the photographs are indicated.

We define a polar bear record as extending from the moment that a bear entered the study area until departure. Typically, bears in the study area also visited the main colony island. A colony visit was composed of one or several periods of uninterrupted foraging (searching for bird nests and eating clutches) alternating with resting periods (bears lying on the ground with head down). Resting took place on the island or on patches of snow on the adjacent tundra. Distinction of sex was based on posture and size^42,66,67^. Females were further assigned to reproductive classes based on whether they were accompanied by cubs or not. Within sex categories, bears were classified by size, which tentatively corresponds to age classes (subadult and adult) and was facilitated by observing bears from a standard position and at similar distances. Whenever possible, polar bears were identified individually based on sex, body shape and size (Fig. 2), and the pattern of whisker spots^68^. Identification was improved by using a set of physiognomic characteristics as derived from photographs taken in the field at distances of up to 400 m^69^. Further support came from information on presence or absence of various types of ear marks^70^, satellite transmitters or numbers painted on the back (supplementary Data Records). On the rare occasions that identification through these methods was not possible, we were generally able to track down the bear’s identity by linking the record to previous or subsequent observations of the same individual (supplementary Data Records). Alternatively, circumstantial evidence was used to ascertain whether the presence of a bear was unique in a particular year, although its identity remained masked. For example, we detected the tracks of a female polar bear with two cubs crossing the study area after a snow storm on 26 May 2019. In this particular year, we did not observe any other polar bear families, which means that this particular female was not observed on other occasions in 2019. Still, it remained unknown whether this female was observed in other years. Another uncertainty was introduced by the growing number of observation years. Repeated observations of individuals over several years indicated that distinctive characteristics might change. As a consequence, individual identification might be hampered if a bear did not show up regularly over the years, potentially leading to an overestimate of the number of different individuals. To arrive at a second, more conservative estimate, we matched any first record of a polar bear individual to previous records of a potentially corresponding group of bears as defined by sex, age class, and absence or presence of markings (details in supplementary Data Records). With any match in the dataset and no evidence to the contrary, such bears were rated as “possibly observed in previous years”.

Intervals between successive individuals were calculated from the period of time between bear records as the difference between the time of arrival in the study area and time of departure of the previous individual. Successive records of the same individual, without other individuals showing up in between, were regarded as a single unit, such that the time of arrival was taken from the first record and the time of departure from the last.

As a measure of walking rate, we recorded the time needed for 15 complete step cycles^71^ during walking gait^72^ to the nearest 0.1 s. We suppose that within sex and age groups, these step times properly reflect variation in walking rates as large quadruped mammals, including polar bear, primarily modify step (or stride) frequency rather than step length to adjust speed^73^. Records were collected opportunistically within the study area, and each record was composed of 10–15 sequential assessments. For sake of comparison, individuals were recorded when walking within 100 m of the shoreline (on the beach or beach wall) and moving in a direction away from the observer.

The time spent in the colony was calculated as the time period from arrival on the colony island up to departure. Foraging activities included feeding, moving, and vigilance behaviour. Occasional resting periods on the mainland, but within the study area, were included in the colony time.

The total number of predated clutches during a visit was established by direct observation. If needed, a correction was applied for the time that bears were out of sight by dividing the time out of sight by the average interval between subsequent nest predation events^56^. To focus analyses on bears potentially deriving nutritional benefits from foraging, records with nest density below an arbitrary threshold of 1 per ha at bear arrival were not considered in analyses.

### Statistical analyses

We used R version 4.2.3 in the RStudio environment for statistical analyses. The package *glmmTMB*^74^ was used to generate generalised linear mixed effects models. The most appropriate statistical distribution of dependent variables was chosen through package *fitdistrplus*^75^. Continuous predictor variables were mean-centred. To account for possible recurrence of individuals within the same season, the structure of random effects was specified as identity of the bears nested within year of observation. As an exception, the structure of random effects in pace rate analyses reflected a design with multiple observations by record nested within year. Random factors were always included in the models, also when explaining close to zero variance. The package *DHARMa*^76^ was used to detect overdispersion or any deviations from the expected distributions. Significance of fixed effects was obtained using Wald tests with associated z-score. To find the most parsimonious model, we started with a global model that included main effects of predictor variables with any relevant interaction terms^77^. As we did not have an a priori hypothesis about which combination of predictor variables would provide the most parsimonious model, a candidate set was generated from all lower order models using the package *MuMIn*^78^. Models containing both sex and reproductive class (which included a sex effect) were not considered. Models were ranked by the Akaike Information Criterion (AICc as corrected for small sample sizes) and were selected when the AICc value was within 2 units of the lowest AICc in the candidate set. Parameter estimates were (full-)averaged across selected models by the R package *MuMIn*^78^. The associated standard errors were unconditional, which accounts for sampling variance and model selection uncertainty^79^. For each model, the conditional and marginal coefficients of determination R^2^ were calculated following Nakagawa et al.^80^ using the package *MuMIn* and interpreted as the variance explained by the fixed effects only and the fixed and random effects combined, respectively^81^. Group means are reported ± standard errors, as obtained from estimated marginal means by package *emmeans*^82^. When several models contributed to a selected set for further model averaging, marginal means were derived from the global model rather than from the averaged model^83^. A Tukey adjustment for multiple comparisons was applied whenever appropriate.

To analyse variation in intervals, a gamma error distribution with a log link was adopted. The global model contained age class and sex of the focal animal, and sex and reproductive class of the previous bear, and up to three-way interactions as fixed effects. To investigate whether the observed distribution of intervals was skewed in any way, we compared the observations with intervals generated based on random timing of arriving. As a first step in each simulation, each of the years’ arrival times were randomised and intervals between consecutive visits were calculated while accounting for an averaged residence time in the study area. To achieve a conservative measure of intervals and avoid bias towards longer intervals, the simulations did not cover the periods beyond the actual field observations. Subsequently, differences in intervals (with 95% confidence bands, based on percentiles) between simulations and field observations were generated by non-parametric bootstrapping (R package *boot*). Average confidence bands of intervals for both sexes were calculated across 5000 randomisations.

To explore associations between the sex of subsequent individuals, the dependent variable was set as the sex of the focal bear as modelled using a binomial error distribution. The global model contained the fixed factors sex and reproductive class of the previous bear, age class of the focal individual, and interval between departure of the previous bear and arrival of the focal individual as covariates, together with three-way interaction terms.

Variation in time needed for 15 steps was analysed using a gamma error distribution with a log link function. The fixed effects part of the global model contained age class and sex of the focal individual, sex of the previous individual, and the time elapsed after departure of the previous individual (represented by “intervals”), together with two-way interactions between sex and each of the other variables.

To explore variation in foraging performance, the adopted error distributions were set to a gamma distribution with a log link function (for the time spent in the colony), a negative binomial distribution using the nbinom2 parameterisation with a log link (for the number of clutches taken), and a binomial link (for the probability of resting in the colony). Predictor variables in the global models were age class and sex of the focal animal, and sex and reproductive class of the previous individual, the time elapsed after the previous individual, and nest density on a log scale, together with two-way interactions between sex and each of the other variables. The observations featured an excess of zero-values because not all individuals in the study area visited the colony. To handle excess zeros in the time and predation models, a zero-inflation component was added to the models. As we expected a seasonal pattern in colony visitation, we started with a second-order polynomial using date as a fixed effect in the zero-inflation part. However, due to convergence problems, we simplified the global models by taking nest density as the fixed effect in the zero-inflation part.

To compare food intake between the sexes, we scaled the number of clutches taken per visit to metabolic requirements as $\left({MM}_{female}/{MM}_{male}\right)\times \left({{BM}_{male}}^{0.75}/{{BM}_{female}}^{0.75}\right).$ The result represents the proportional amount of energy needs covered by females relative to those by males. MM represents the marginal means of the global model for average nest density and average interval between successive records. Marginal means were averaged across the sex of the previous individual, and were conditional on visiting the colony. We followed Pagano et al.^84^ in that metabolic requirements were set as proportional to body mass (BM) raised to the power of 0.75. Average body mass was set at 389 kg and 185 kg for adult males and females, respectively, and for subadults at 257 kg and 138 kg^31^, respectively.

### Ethics statement

The study complied with relevant national and international guidelines and legislation. Permissions to conduct the study and fieldwork were obtained each year from the Governor of Svalbard (RiS-ID 3533).

## Results

The average annual numbers of polar bears visiting the study area (from the end of May through the end of July) were 5.5 females (range 2–11) and 4.7 males (range 1–10). The average numbers of different individuals annually were 2.8 females (range 2–5) and 2.8 males (range 1–4). Over the 14 years of this study, 11–14 different females and 14–21 males were identified (supplementary Data Records).

### Intervals between successive bears

Intervals between successive bears in the study area varied between 0 days (when a bear arrived while the previous individual was still in the area, which occurred six times) and 32.4 days. The frequency distribution of intervals peaked at values of less than one day with a skew to the right (supplementary Fig. S1), and 50% of the intervals were shorter than 2.25 days (n = 91). Models containing sex and reproductive class of the previous individual had little statistical support (supplementary Table S1). The length of intervals varied by sex of the focal individual, with a weak additional effect of age class (Table 1, supplementary Table S1). Females came 4.6 days (± 1.06) after a previous bear, and males after 2.7 days (± 0.64). The observation that males succeeded other bears more closely than females was further supported by bootstrapping. Randomising the timing of bear arrivals across the seasons and sexes resulted in intervals of on average 5.2 days (± 0.03). The observed average interval between females and a previous bear did not differ from the simulated values (on average 0.05 days shorter, 95% confidence bands: –2.68, 2.78), whereas intervals between males and a previous individual were significantly shorter than the simulated values (on average 2.27 days less, 95% confidence bands: 0.48, 4.06).

**Table 1:** Intervals (days) between arrival of successive polar bears in the study area.

Predictor	Estimate	SE	Z-score	*P*
Intercept	1.14	0.214	5.342	< 0.001
Sex	0.63	0.292	2.150	0.032
Age	− 0.14	0.268	0.516	0.606

Model-averaged parameter estimates of the fixed parts of selected mixed effects models. Estimates are on the log scale. Predictor variables are sex and age class (Age) of focal animal. Random effects in the model are random intercepts of individual identity nested within year. Reference categories are male in sex, and adult in age class. Estimates were obtained from 91 records across 12 years and 64 year × individual combinations. See supplementary Table S1 for model selection.

### Sequence by sex

**Fig. 3:**
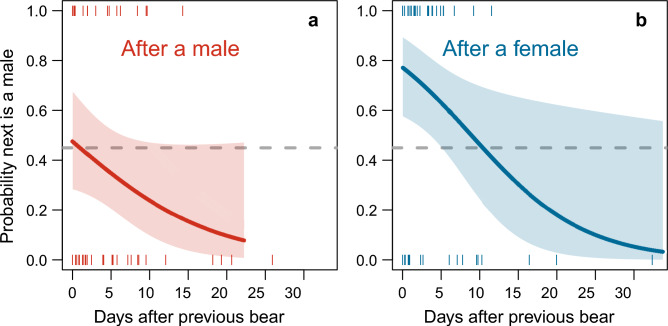
Probability that the focal (next) individual is a male in relation to the time elapsed after departure of a polar bear. Lines represent the predictions of the averaged mixed effects model given in Table 2 with 95% confidence bands, after departure of a male (**a**) or a female (**b**), and averaged across age classes. The horizontal dashed line represents the averaged proportion of males in the local observations (0.46, n = 107).

In 63% of the records (95% confidence band: 52.4%, 71.9%), bears were succeeded by individuals of the opposite sex. The selected models exploring patterns in sequence contained the interval between successive records, age class of the focal individual, and sex of the previous bear in interaction with interval (Table 2, supplementary Table S2). Notably, during the first five days after a female departed from the study site, successors were dominantly males (Fig. 3) —either adults or subadults— with the odds ratio in favour of males starting at 3.3 on the first day after departure of the female, and thereafter dropping daily by a factor 0.90 (as obtained by exponentiating the interval effect, Table 2). After departure of a male, new arrivals were female-biased with an initial odds ratio of 1.1, which increased to 9 after 20 days. However, the 95% confidence bands of the estimates overlapped with the average proportion of males in the local population, which means that the pattern was not significantly different from a random pattern (Fig. 3). The weak age effect (Table 2), together with lack of support for the interaction term between age and sex of the previous individual (supplementary Table S2), indicates that a preference for either sex was irrespective of age.

**Table 2:** Probability that the sex of the next (focal) polar bear is male.

Predictor	Estimate	SE	Z-score	*P*
Intercept	− 0.56	0.371	1.503	0.133
Age	− 0.12	0.332	0.347	0.729
Interval	− 0.11	0.069	1.541	0.123
PrevSex	1.19	0.515	2.302	0.021
Interval × PrevSex	− 0.03	0.077	0.389	0.698

Model-averaged parameter estimates of the fixed parts of selected mixed effects models. Estimates are on the log-odds scale. Predictor variables are sex of the previous individual (PrevSex), age class of the focal individual (Age), and the number of days elapsed after the previous bear (Interval). The reference category is male in PrevSex, and adult in Age. Interactions are indicated by ‘ × ’. Random effects in the model are random intercepts of individual identity nested within year. Estimates were obtained from 91 records across 12 years and 68 year × individual combinations. See supplementary Table S2 for model selection.

### Rate of movement

**Fig. 4:**
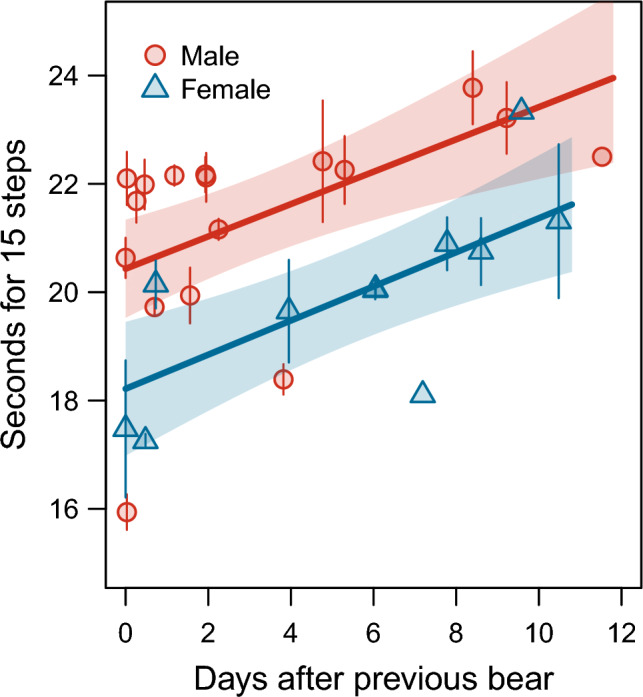
Step times of male and female polar bears walking along the coast line (seconds per 15 step cycles) in relation to the time elapsed after a previous individual. Given values are means per record with 95% confidence bands. Lines represent the predictions for adult males and females of an averaged mixed effects model (Table 3) with 95% confidence bands. Note that the longer step times in males may be compensated by larger step lengths.

The selected models explaining variation in step times contained sex, age class, and interval between successive records (Table 3, supplementary Table S3). In addition to the longer step times in males than in females, step times increased with increasing time elapsed after a previous bear (Fig. 4). Step times tended to be shorter in subadults than in adults, but a small sample size of subadults led to low statistical power of this result.

**Table 3:** Step times of polar bears walking along the coast line in the study area (seconds per 15 steps).

Predictor	Estimate	SE	Z-score	*P*
Intercept	21.94	0.441	49.784	< 0.001
Sex	− 2.13	0.605	3.528	< 0.001
Age	− 1.30	0.885	1.469	0.142
Interval	0.30	0.083	3.586	< 0.001
Age × Sex	0.42	1.011	0.418	0.676
Interval × Sex	0.02	0.076	0.250	0.803

Model-averaged parameter estimates of fixed part of the selected mixed effects models with predictor variables sex, age class, and the number of days elapsed after the previous individual (Interval). Male is the reference category in sex, and adult is the reference category in age class. Interactions are indicated by ‘ × ’. Random effects in the model are observation record nested within year of observation. Estimates obtained from 305 observations across 35 records and 14 individual polar bears. See supplementary Table S3 for model selection.

### Foraging performance in colony

**Fig. 5:**
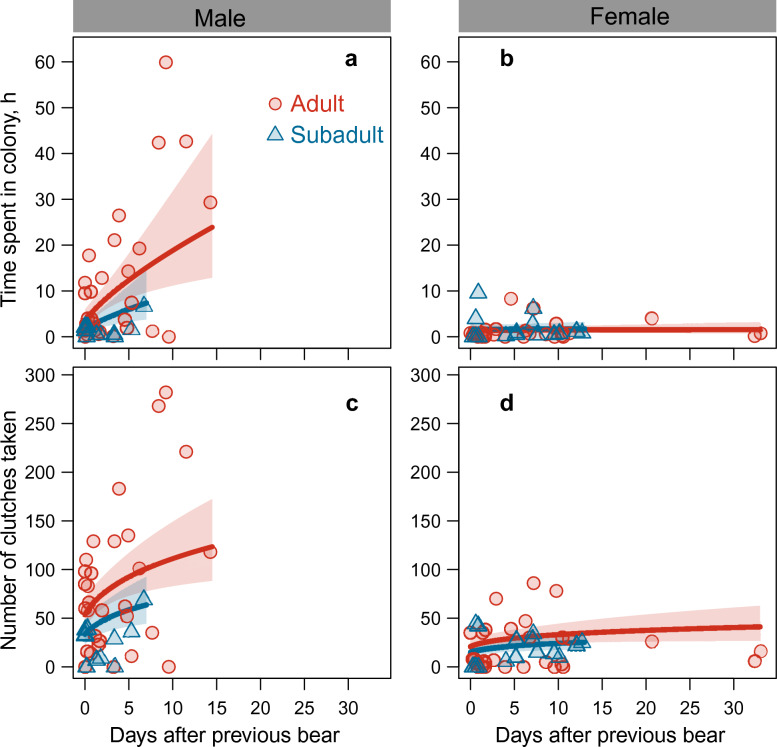
Polar bear behaviour in the study area in relation to the time elapsed after a previous individual. Given are (**a**, **b**) the number of hours spent in the bird colony, and (**c**, **d**) number of clutches taken for adult and subadult males (left panels) and females (right panels). Lines represent the predictions of the averaged mixed effects models given in Tables 4 and 5 with 95% confidence bands, averaged across sex of the previous individual, if appropriate, and for an average nest density. Shown are the raw data.

The time that polar bears spent in the bird colony varied by sex, age class, the interval between successive records, and density of nests (Table 4, supplementary Table S4). Adult males spent on average 8.0 (± 1.44) hours in the colony (conditional on visiting the colony), which was considerably longer than residence times of adult females (1.6 ± 0.33 h), subadult males (3.0 ± 0.99 h), and subadult females (1.9 ± 0.57 h) (all comparisons with adult males z-score > 2.62, *P* < 0.04). Models containing the sex of the previous individual had little support (supplementary Table S4). With increasing number of days elapsed after departure of the previous individual, males —but not females— spent more time in the colony (Fig. 5). Further, both males and females spent more time in the colony with increasing nest density. The zero-inflated part of the model showed that at an average density 15.6% of the bears (obtained by converting the log-odds of the intercept, Table 4) passed by without visiting the colony.

**Table 4:** Time (h) spent by polar bears in the bird colony.

Predictor	Estimate	SE	Z-score	*P*
A. Conditional part				
Intercept	2.07	0.193	10.722	< 0.001
Sex	− 1.56	0.303	5.156	< 0.001
Age	− 0.71	0.574	1.230	0.219
Interval	0.66	0.179	3.692	< 0.001
Density	0.48	0.086	5.551	< 0.001
Age × Sex	0.84	0.716	1.175	0.240
Interval × Sex	− 0.63	0.247	2.569	0.010
B. Zero-inflation part				
Intercept	− 1.69	0.331	5.094	< 0.001
Density	0.40	0.339	1.170	0.242

Model-averaged parameter estimates of the fixed part of selected mixed effects models with predictor variables sex, age class, the number of days elapsed after the previous individual (Interval, log transformed), density of nests (Density, log transformed) The model is separated in (A) a conditional part (conditional on visiting the colony, on a log scale), and (B) a zero-inflation part (modelling the probability not to visit the colony, on a log-odds scale). Male is the reference category in sex, and adult is the reference category in age class. Interactions are indicated by ‘ × ’. Random effects in the model are random intercepts of individual identity nested within year. Estimates were obtained from 87 records across 12 years, and 54 individual × year combinations. See supplementary Table S4 for model selection.

Variation in the number of predated clutches was associated with sex, age class, interval between successive records, nest density, and sex of the previous individual (Table 5, supplementary Table S5). With increasing number of days elapsed after departure of the previous individual, polar bears took more clutches (Fig. 5), a trend that was more prominent in males than in females. On average, adult males took 79.3 (± 9.99) clutches during a visit (conditional upon taking any clutch at all), which was considerably more than the number of clutches taken by adult females (25.2 ± 3.63), subadult males (39.9 ± 9.80), and subadult females (22.8 ± 5.65) (all comparisons with adult males z-score > 2.54, *P* < 0.05). Females preceded by another female tended to ingest fewer clutches than females coming after a male (17.7 ± 3.41 and 26.6 ± 2.92, respectively, z = 1.89, *P* = 0.059).

**Table 5:** Number of clutches taken by polar bears during visits to the bird colony.

Predictor	Estimate	SE	Z-score	*P*
A. Conditional part				
Intercept	4.10	0.128	32.010	< 0.001
Sex	− 0.66	0.190	3.492	< 0.001
Age	− 0.39	0.171	2.313	0.021
Interval	0.26	0.083	3.191	0.001
Density	0.57	0.083	6.862	< 0.001
PrevSex	0.10	0.157	0.630	0.528
PrevSex × Sex	− 0.56	0.328	1.708	0.088
Age × Sex	0.04	0.140	0.259	0.796
Interval × Sex	− 0.06	0.117	0.482	0.630
Density × Sex	− 0.06	0.100	0.581	0.561
B. Zero-inflation part				
Intercept	− 1.86	0.395	4.728	< 0.001
Density	0.75	0.361	2.074	0.038

Model-averaged parameter estimates of the fixed part of selected mixed effects models with predictor variables sex, age class, sex of previous individual, the number of days elapsed after the previous individual (Interval), and density of nests (Density). The model is separated in (A) a conditional part (conditional on visiting the colony and eating eggs, on a log scale), and (B) a zero-inflation part (modelling the probability not to visit the colony and eating eggs, on a log-odds scale). Further details in Table 4. See supplementary Table S5 for model selection.

While adjusting for differences in metabolic body mass, females ingested on average only 58.7% (95% confidence band 38.8%–89.0%) of the males’ intake during a colony visit. In subadults, differences between the sexes were smaller with females ingesting on average 72.7% (35.4%–149.5%) of the number of clutches taken by males.

**Fig. 6:**
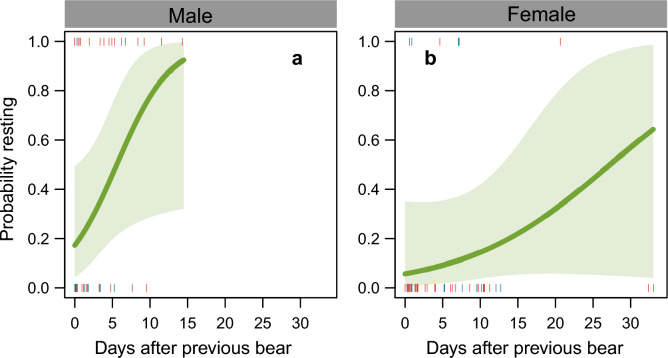
The probability of resting in the colony for (**a**) males and (**b**) females in relation to the time elapsed after a previous individual. Lines represent the predictions of the averaged mixed effects models given in Table 6 with 95% confidence bands, for an average nest density.

The probability of a polar bear resting in the colony or adjacent tundra varied by sex, interval between successive records, and nest density (Table 6, supplementary Table S6). In males, the probability of resting increased with time elapsed after a previous bear, whereas in females, the probability was consistently low (Fig. 6). On average, males were more likely to rest in the colony than females, with the odds for males being 7.5 times higher than for females (Table 6).

**Table 6:** Probability that a polar bear takes a rest break in the study area, including the bird colony.

Predictor	Estimate	SE	Z-score	*P*
Intercept	− 0.21	0.632	0.341	0.733
Sex	− 2.02	0.962	2.096	0.036
Interval	0.24	0.165	1.451	0.147
Density	0.96	0.419	2.300	0.021
Interval × Sex	− 0.15	0.174	0.842	0.400

Model-averaged parameter estimates of the fixed part of selected mixed effects models with predictor variables sex, the number of days elapsed after the previous individual (Interval), and the density of nests (Density). Estimates are on the log-odds scale. Further details in Table 4. See supplementary Table S6 for model selection.

## Discussion

Polar bear densities are among the lowest among carnivores^85–87^, yet polar bears are known to exhibit an interest in each other for much of the year^37,57,88^. This study confirms persistence of sex-mediated travel patterns after the mating season, and suggests that foraging decisions include a trade-off between food intake and getting closer to others (in males) or maintaining safe distances (in females). Travel patterns of male polar bears corresponded to the strategy of closing the proximity gap. Male polar bears followed conspecifics, in particular females, more often and more closely than expected if individuals move independently of one another. Females tended to follow males, however without being particularly close to males, and the sequence could have arisen from males occupying positions immediately after females rather than from females actively seeking the presence of males.

The disparate behaviour between the sexes in the bird colony suggests that males and females balanced the benefits and potential costs of foraging in different ways. When males were close to other bears, they walked faster and reduced residence time in the colony, including rest time. Although we do not exclude the possibility of alternative explanations, these behaviours are consistent with a strategy to keep up with an individual that one is following^14^, and are among the behavioural tools used to increase probability of encountering other individuals^14,38^. This supports the hypothesis that, when conditions for contact were favourable, males prioritised maintaining a link to others over gaining energy. Female residence times in the colony were considerably shorter than those of males and, notably, it was uncommon to see females resting in the colony. Instead, females departed from the colony before initiating a resting bout. This corresponds to behaviours observed in other carnivores when other predators are nearby. For example, female cheetahs (*Acinonyx jubatus*) retreated sooner from their prey after a meal when lions (*Panthera leo*) were present, even when they ran the risk of losing their unguarded kill to scavengers^89^. Likewise, leopards (*Panthera pardus*) were found to rest at greater distances from a kill when lions or competing male conspecifics were present^22^. Choosing where to spend a resting break, at the kill site itself or at some distance, includes a trade-off between the costs and benefits of each option^24^. When the risk increases of predators being attracted to the kill site, the choice will be to seek a safer place. It seems reasonable to assume that female polar bears run less risk of encountering males on the vast expanses of the tundra than within the limited confines of bird breeding colonies. Therefore, female choice of lower-risk resting sites supports the hypothesis that females followed a strategy of avoiding males. This is further supported by the observation that females increased walking speed, thus reducing the time needed to reach a refuge, when other bears were nearby.

Investing in familiarity with a potential mating partner during the non-breeding season may be particularly important in male polar bears because access to females is subject to strong competition^33,39^. Because of strong intra-sex competition^39^, lifetime reproductive success of male polar bears may be extremely skewed, and in one population only 5% of the males sired more than 50% of the offspring^39^. The adaptive value of investing in successful reproduction beyond the breeding season might therefore be high. Building on familiarity with potential mates is a strategy to enhance reproductive success in a wide variety of species^8,40,90–92^. Familiarity enhances reproductive success by positive effects on compatibility between partners^93^, a safer environment to rear offspring^94^, shortening the period to find a partner^95^, or appeasement among partners that could potentially harm each other^96^. Building up familiarity may be markedly important in promiscuous species, such as the polar bear^63,97^, because promiscuous females tend to avoid males with which they have already mated^98^. We propose that the spatial and behavioural patterns observed in this study reflect the process of male polar bears attempting to get acquainted with members of the opposite sex or to improve their knowledge of others. Further work is needed to establish the efficacy of this strategy.

Despite a male bear’s intentions, an approaching male polar bear is a potential threat to females. Apart from the possibility of males killing cubs or females, males and females are competitors for food when visiting the same food patch and males might respond accordingly^99^. Females are not necessarily subordinate to males^32^ but when it comes to aggressive interactions, the smaller females are at a disadvantage and are likely to be injured^33,66,100^. An option of sexual spatial segregation^101^ is subject to suitability of geography and distribution of food resources^102,103^. Suitable conditions may be unavailable to polar bears in parts of the Arctic, including Svalbard, and possibly aggravated by declining sea ice cover and shrinking availability of habitat. In fact, the behaviours that we described may have been amplified by the polar bears’ shift to a land-based lifestyle along this coastline in Svalbard. Although polar bear distribution on sea ice is extremely patchy^86,104,105^, opportunities to avoid others might be particularly reduced when residing on land strips in a landscape free of sea ice.

Most of the individuals in our study were seen during several years (supplementary Data Records) but, apart for the associations of mothers and their 0- and 1-year old offspring, we did not detect apparent links between individuals. However, we suspect that many of the observed bears shared a common winter range in nearby fjords, with possibly overlapping tracks. Polar bears do not form networks as observed in several other carnivores^21^, but the bears in our study may have had common histories, with behavioural dependencies and relationships among individuals beyond the mother–offspring bonds^106^. With current knowledge, it is difficult to assess to what extent these histories may have affected our results. Formation of networks may become more apparent with a shrinking polar bear population or when populations become isolated due to loss of sea ice^13,107^.

Behaviour of subadults was not strikingly different from that of adults, which indicates that young polar bears may be under similar adaptive pressures as adults. Getting acquainted with others, learning from others, and avoiding fatal encounters are probably as important in subadults as in adults, although the time scale of the pay-off may be different. However, subadults may also form semi-social bonds with siblings or their mothers which are lost at an older age. During the first months after family break-up, siblings may team up together, following each other or their mother at a distance. Thus, this family behaviour may have contributed to some of the trends observed in this study.

The divergent strategies in male and female polar bears, with males benefitting from proximity to others and females from keeping distance, suggest a conflict between the sexes over proximity. Sexual conflicts are often associated with events during the breeding period, such as those over mating rate, parental effort, or clutch size^108^. However, conflicts may arise outside the breeding season as well. For example, in brown bears (*Ursus arctos*), females preferring sub-optimal habitat, closer to humans but void of aggressive males, were more successful in rearing their cubs than females in optimal habitat^103^. The role of intra-specific distances and the adjustments of proximity occupy a central place in the study of population processes^109^. However, it is uncommon that in this context proximity may also be a source of conflict between the sexes. We suggest that in solitary carnivores, opposing interests to proximity in the sexes may be an important aspect of the mating system.

In the process of coping with divergent strategies, polar bears may sacrifice foraging time and food intake for the benefit of future reproductive success (in males) or survival (in females). Thus, male residence time in the colony dropped with proximity to other polar bears. Over the range of proximities observed (from 0 to 14 days), the residence times varied by a factor of five, while other variables were kept constant (Fig. 5). We interpret this finding to suggest that males reduced residence times by up to 80% as a response of proximity to others. This had an impact on food intake and over a similar range of proximities, the number of clutches taken varied by a factor of two, corresponding to a reduction by up to 50% (Fig. 5). Males were apparently able to partially compensate for shorter residence times by taking more clutches per unit of foraging time. Nevertheless, the reduction in food intake over the range of proximities remained considerable. Similarly, we interpret the reduced food intake by females –representing only half of the intake by males under average food conditions– as an energy cost emerging from an inter-sexual conflict. Both these effects are biologically significant because energy intake during spring and summer must provide the body stores needed in the subsequent winter^34^. Moreover, food conditions for land-based polar bears are likely to be inferior to food resources available to polar bears on sea ice^87,110,111^, which is additional to any existing nutritional stress^112,113^. Females could possibly mitigate energy consequences of brief visits by returning more often to the colony, which they did in 53% of the cases (n = 34) (versus 29% in males, n = 34, χ^2^(1) = 2.98, *P* = 0.085). Nevertheless, by spending more time walking to and from resting places away from the bird colony, females inevitably lost energy and time, and moreover foraging opportunities were lost if another bear had visited the colony in the meantime.

Our study indicates that investing in successful reproduction may include spatial arrangements, as a constant process with consequences for both sexes. This result supports those of Laidre et al.^38^ who showed that local polar bear populations exhibit a spatial structure due to non-random patterns in positioning and movements by both of the sexes relative to each other. Our study adds to their findings in that we found these patterns need not be restricted to the mating season but may occur during other times of the year. Moreover, we provide evidence that the interactions between the sexes have an impact on foraging behaviour and food intake, which are important parameters for a species with high energy needs and quickly changing habitats.

### Supplementary Information

Supplementary Information.

## Data Availability

Datasets used during the current study are included in this paper and its Supplementary Information file, or are available from the corresponding author on reasonable request.
