# Supplementary material for: Land-based foraging by polar bears reveals sexual conflict outside mating season
Source: Sci Rep. 2024 Aug 31;14:20275. doi: 10.1038/s41598-024-71258-w (PMC11365984; doi:10.1038/s41598-024-71258-w)
Supplement: Supplementary file 1 — Supplementary Information. [file 41598_2024_71258_MOESM1_ESM.pdf]

Supplementary information

Land-based foraging by polar bears reveals sexual conflict outside mating season

Authors: Jouke Prop, Jeffrey M. Black, Jon Aars, Thomas Oudman, Eva Wolters, Børge Moe

This file contains:

Supplementary Figure S1

Supplementary Table S1

Supplementary Table S2

Supplementary Table S3

Supplementary Table S4

Supplementary Table S5

Supplementary Table S6

Supplementary Data Records

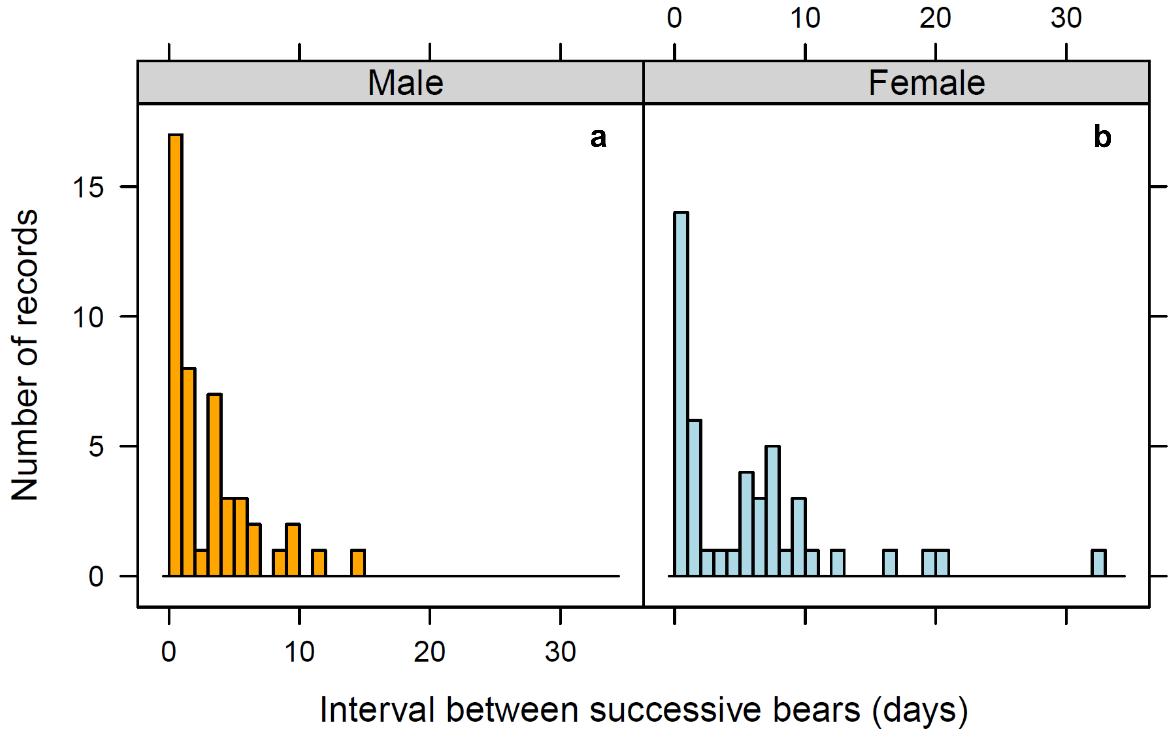

Figure S1. Frequency distributions of the intervals between departure from the core study area of a polar bear and arrival of the focal individual. Panel (a) gives data for males, and panel (b) for females. Data were collected in 2009–2022. Intervals were categorized by multiples of one day.

Table S1. Candidate set of models for glmm (R function glmmTMB adopting a gamma distribution with log-link) examining variation in the **length of intervals between successive polar bears** in the core study area. Fixed factors were age (Age) and sex of the focal animal (Sex), and sex (PrevSex) and reproductive class (male, female without cubs, female with cubs) of the previous individual (PrevRepro). Random intercepts included individual identity nested within year of observation. Given for each model are the number of parameters (K), the log-likelihood (logLik), AICc corrected for small sample sizes, the difference in AICc relative to the model with the lowest AICc (Delta), model probability (Weight), and the marginal (R2marg) and conditional (R2cond) coefficient of determination. Interactions are indicated by '×'. The models are ordered by increasing AICc. Only the first ten top-ranking models are shown. Weight is based on the complete list of candidate models. The global model contained the following terms: Age×PrevSex×Sex + Age×PrevRepro×Sex.

Model	K	logLik	AICc	Delta	Weight	R2marg	R2cond
Sex	5	−204.17	419.04	0.000	0.251	0.084	0.084
Age + Sex	6	−203.54	420.08	1.044	0.149	0.105	0.105
Intercept only	4	−206.36	421.19	2.153	0.086	0.000	0.029
PrevSex + Sex	6	−204.16	421.31	2.274	0.081	0.082	0.082
PrevSex + Sex + PrevSex×Sex	7	−203.06	421.48	2.441	0.074	0.116	0.116
Age + Sex + Age×Sex	7	−203.54	422.43	3.393	0.046	0.105	0.105
Age + PrevSex + Sex	7	−203.54	422.43	3.393	0.046	0.105	0.105
Age	5	−205.96	422.62	3.583	0.042	0.018	0.026
PrevRepro + Sex	7	−203.97	423.28	4.246	0.030	0.094	0.094
Age + PrevSex + Sex + PrevSex×Sex	8	−202.81	423.37	4.337	0.029	0.119	0.119

Table S2. Candidate set of models for glmm models (R function glmmTMB adopting a binomial distribution) examining variation in the **probability that the focal polar bear is a male**. Fixed factors were age (Age) of the focal animal, and sex (PrevSex) and reproductive class (male, female without cubs, female with cubs) of the previous individual (PrevRepro). Covariate was the number of days between successive individuals (Interval). Random intercepts included individual identity nested within year of observation. Given for each model are the number of parameters (K), log-likelihood, AICc corrected for small sample sizes, difference in AICc relative to the model with the lowest AICc (Delta), model probability (Weight), and the marginal (R2marg) and conditional (R2cond) coefficient of determination. Interactions are indicated by '×'. The models are ordered by increasing AICc. Only the first ten top-ranking models are shown. Weight is based on the complete list of candidate models. The fixed part of the global model contained the following terms: PrevRepro×Interval×Age + PrevSex×Interval×Age.

Model	K	logLik	AICc	Delta	Weight	R2marg	R2cond
Interval + PrevSex	5	−56.78	124.27	0.000	0.244	0.184	0.216
Interval + PrevSex + Interval×PrevSex	6	−56.27	125.54	1.267	0.130	0.210	0.239
Interval + Age + PrevSex	6	−56.36	125.72	1.451	0.118	0.197	0.231
Interval + PrevRepro	6	−56.72	126.45	2.177	0.082	0.189	0.228
Interval + Age + PrevSex + Interval×PrevSex	7	−55.77	126.90	2.627	0.066	0.225	0.256
Interval + Age + PrevRepro	7	−56.27	127.89	3.618	0.040	0.204	0.249
Interval + Age + PrevSex + Age×PrevSex	7	−56.29	127.92	3.653	0.039	0.199	0.235
Interval + Age + PrevSex + Interval×Age	7	−56.32	128.00	3.729	0.038	0.196	0.228
Interval	4	−60.00	128.47	4.199	0.030	0.103	0.103
PrevSex	4	−60.06	128.58	4.313	0.028	0.085	0.111

Table S3. Selection results for glmm models (R function glmmTMB) examining variation in the **seconds for 15 step cycles by polar bear** while travelling in the core study area. Fixed factors were age (Age) and sex (Sex) of the focal individual, and sex of the previous individual (PrevSex). Covariate was the number of days between successive individuals (Interval). Random effect in all models was observation record nested within year. Given for each model are the number of parameters (K), Log-likelihood, AICc corrected for small sample sizes, difference in AICc relative to the model with the lowest AICc (Delta), model probability (Weight), and the marginal (R2marg) and conditional (R2cond) coefficient of determination. Interactions are indicated by '×'. The models are ordered by increasing AICc. Only the first ten top-ranking models are shown. Weight is based on the complete list of candidate models. The fixed part of the global model contained the following terms: PrevSex×Sex + Interval×Sex + Age×Sex.

Model	K	logLik	AICc	Delta	Weight	R2marg	R2cond
Age + Interval+ Sex	7	−483.90	982.17	0.000	0.207	0.335	0.768
Age + Interval+ Sex + Age×Sex	8	−483.26	983.01	0.845	0.136	0.355	0.769
Age + Interval+ Sex + Interval×Sex	8	−483.65	983.79	1.619	0.092	0.333	0.764
Interval + Sex	6	−485.90	984.07	1.904	0.080	0.300	0.776
Age + Interval+ PrevSex+ Sex	8	−483.89	984.27	2.100	0.072	0.335	0.768
Age + Interval+ Sex + Age×Sex + Interval×Sex	9	−482.95	984.51	2.339	0.064	0.359	0.767
Age + Interval+ PrevSex+ Sex + PrevSex×Sex	9	−483.11	984.83	2.659	0.055	0.381	0.778
Age + Interval+ Sex + Interval×Age + Age×Sex	9	−483.26	985.14	2.967	0.047	0.354	0.769
Age + Interval+ PrevSex + Sex + Age×Sex + PrevSex×Sex	10	−482.22	985.19	3.022	0.046	0.397	0.776
Age + Interval+ PrevSex+ Sex + Interval×Sex	9	−483.65	985.91	3.743	0.032	0.333	0.764

Table S4. Selection results for glmm models (R function glmmTMB) examining variation in the **time spent in the bird colony by polar bears**. Fixed factors were age and sex of the focal individual, and sex (PrevSex) and reproductive class (male, female without cubs, female with cubs) of the previous individual (PrevRepro). Covariates were the number of days between successive individuals (Interval). and nest density. Random intercepts included were individual identity nested within year of observation. The models contained a zero-inflation part (zi(Density)). Given for each model are the number of parameters (K), Log-likelihood, AICc corrected for small sample sizes, difference in AICc relative to the model with the lowest AICc (Delta), model probability (Weight), and the marginal (R2marg) and conditional (R2cond) coefficient of determination. Interactions are indicated by '×'. The models are ordered by increasing AICc. Only the first ten top-ranking models are shown. Weight is based on the complete list of candidate models. The fixed part of the global model contained the following terms: PrevSex×Sex + PrevRepro×Sex + Interval×Sex + Density×Sex + Age×Sex + Zi(Density).

Model	K	logLik	AICc	Delta	Weight	R2marg	R2cond
Age + Interval + Density + Sex + Age×Sex + Interval×Sex + Zi(Density)	12	−185.68	399.59	0.000	0.237	0.656	0.656
Interval + Density + Sex + Interval×Sex + Zi(Density)	10	−189.11	401.11	1.529	0.110	0.632	0.659
Age + Interval + Density + Sex + Age×Sex + Interval×Sex + Density×Sex + Zi(Density)	13	−185.54	402.07	2.485	0.068	0.656	0.656
Age + Interval + Density + prevSex + Sex + Age×Sex + Interval×Sex + Zi(Density)	13	−185.67	402.33	2.745	0.060	0.657	0.657
Age + Interval + Density + Sex + Interval×Sex + Zi(Density)	11	−188.44	402.41	2.820	0.058	0.632	0.660
Interval + Density + Sex + Interval×Sex + Density×Sex + Zi(Density)	11	−188.61	402.74	3.156	0.049	0.636	0.664
Age + Interval + Density + Sex + Age×Sex + Zi(Density)	11	−188.84	403.20	3.611	0.039	0.640	0.640
Interval + Density + PrevSex + Sex + Interval×Sex + Zi(Density)	11	−189.09	403.70	4.119	0.030	0.635	0.658
Age + Interval + Density + Sex + Age×Sex + Interval×Sex	11	−189.23	403.99	4.399	0.026	0.656	0.656
Age + Interval + Density + Sex + PrevSex + PrevSex×Sex + Interval×Sex + Density×Sex + Zi(Density)	14	−185.27	404.38	4.797	0.022	0.661	0.661

Table S5. Selection results for glmm models (R function glmmTMB) examining variation in **number of clutches taken by polar bear** in the study bird colony. Fixed and random effects, and zero-inflation part as in supplementary Table S4. Given for each model are the number of parameters (K), Log-likelihood, AICc corrected for small sample sizes, difference in AICc relative to the model with the lowest AICc (Delta), model probability (Weight), and the marginal (R2marg) and conditional (R2cond) coefficient of determination. Interactions are indicated by '×'. The models are ordered by increasing AICc. Only the first ten top-ranking models are shown. Weight is based on the complete list of candidate models. The fixed part of the global model contained the following terms: PrevSex×Sex + PrevRepro×Sex + Interval×Sex + Density×Sex + Age×Sex + Zi(Density).

Model	K	logLik	AICc	Delta	Weight	R2marg	R2cond
Age + Interval + Density+ PrevSex + Sex + PrevSex×Sex + Zi(Density)	12	–339.39	707.00	0.000	0.112	0.758	0.797
Age + Interval + Density+ PrevSex + Sex + Density×Sex + PrevSex×Sex + Zi(Density)	13	–338.58	708.15	1.145	0.063	0.760	0.798
Age + Interval + Density+ PrevSex + Sex + Interval×Sex + PrevSex×Sex + Zi(Density)	13	–338.85	708.69	1.685	0.048	0.758	0.797
Age + Interval + Density+ PrevSex + Sex + Age×Sex + PrevSex×Sex + Zi(Density)	13	–338.90	708.79	1.791	0.046	0.758	0.797
Interval + Density+ Sex + Age + Interval×Sex + Zi(Density)	11	–341.80	709.12	2.115	0.039	0.729	0.774
Age + Interval + Density+ Sex + Interval×Sex + Age×Sex + Zi(Density)	12	–340.47	709.15	2.147	0.038	0.737	0.780
Age + Interval + Density+ Sex + Zi(Density)	10	–343.24	709.37	2.369	0.034	0.724	0.781
Age + Interval + Density+ Sex + Density×Sex + Interval×Sex + Zi(Density)	12	–340.61	709.44	2.441	0.033	0.735	0.778
Age + Interval + Density+ Sex + Density×Sex + Zi(Density)	11	–342.07	709.65	2.652	0.030	0.727	0.776
Interval + Density+ Sex + Interval×Sex + Density×Sex + Zi(Density)	11	–342.07	709.66	2.658	0.030	0.727	0.773

Table S6. Selection results for glmm models (R function glmmTMB) examining variation in the **probability of polar bears taking a rest** in the study bird colony and adjacent tundra. Fixed and random effects as in supplementary Table S4. Given for each model are the number of parameters (K), Log-likelihood, AICc corrected for small sample sizes, difference in AICc relative to the model with the lowest AICc (Delta), model probability (Weight), and the marginal (R2marg) and conditional (R2cond) coefficient of determination. Interactions are indicated by '×'. The models are ordered by increasing AICc. Only the first ten top-ranking models are shown. Weight is based on the complete list of candidate models. The fixed part of the global model contained the following terms: PrevSex×Sex + PrevRepro×Sex + Interval×Sex + Density×Sex + Age×Sex.

Model	K	logLik	AICc	Delta	Weight	R2marg	R2cond
Interval + Density + Sex + Interval×Sex	7	−38.70	92.81	0.000	0.126	0.415	0.564
Interval + Density + Sex	6	−40.23	93.50	0.693	0.089	0.400	0.557
Density + Sex	5	−42.20	95.14	2.326	0.039	0.300	0.440
Age + Interval + Density + Sex + Age×Sex	8	−38.65	95.15	2.343	0.039	0.478	0.552
Interval + Density + Sex + Interval×Sex + Density×Sex	8	−38.69	95.22	2.407	0.038	0.404	0.553
Interval + Density + PrevSex + Sex + Interval×Sex	8	−38.69	95.23	2.418	0.038	0.416	0.562
Age + Interval + Density + Sex + Interval×Sex	8	−38.70	95.24	2.425	0.037	0.415	0.564
Interval + Density + Sex + Density×Sex	7	−40.08	95.57	2.757	0.032	0.471	0.627
Age + Interval + Density + Sex + Age×Sex + Interval×Sex	9	−37.64	95.61	2.802	0.031	0.479	0.569
Interval + Density + prevSex + Sex	7	−40.13	95.68	2.865	0.030	0.404	0.537

Data Records. Records of polar bears, in core study site Nordenskiöldkysten, 2009–2022. Details are given for each of the records.

Column	Description
• Code record	Code of record
• indivID	ID number of individual
• Rank by individual	i^{th} occurrence of individual (irrespective of year)
• Year	Year of observation
• Date of arrival	Date of arrival in core study area (dd-mm-yyyy)
• Number of cubs	Number of associated cubs
• Age class	Age class (subadult / adult)
• Sex class	Sex class (female / male)
• Tags	Presence of tags (collar) or painted number (#nn)
• Ear marks	Presence of ear marks
• NPI code	ID number of individual as used by Norwegian Polar Institute (NPI)
• Quality photographs	Quality of photographs: 0=not available, 1=poor, insufficient for details, 2=reasonable for details, 3=good for details
• Comments	Additional comments

Continued on next page.

Code record	indivID	Rank by individual	Year	Date of arrival	Number of cubs	Age class	Sex	Tags	Ear marks	NPI code	Quality photographs	Comments
2009-01	1	1	2009	27-06-2009	0	adult	M		no		3	
2009-02	2	1	2009	07-07-2009	0	subadult	F				0	Observed more closely the next day
2009-03	2	2	2009	08-07-2009	0	subadult	F		yes		2	
2009-04	3	1	2009	09-07-2009	0	adult	F		yes		1	
2009-07	4	1	2009	17-07-2009	0	adult	F		no		2	
2009-08	5	1	2009	17-07-2009	0	subadult	M		no		1	
2009-09	6	1	2009	19-07-2009	0	adult	M		no		2	
2009-10	2	3	2009	25-07-2009	0	subadult	F		yes		2	
2009-11	3	2	2009	25-07-2009	0	adult	F		yes		2	
2010-01	7	1	2010	09-06-2010	0	adult	M		no		3	
2010-02	1	2	2010	18-06-2010	0	adult	M		no		3	
2010-03	8	1	2010	03-07-2010	0	adult	M		yes		3	
2010-04	2	4	2010	09-07-2010	0	subadult	F		yes		2	
2010-05	8	2	2010	09-07-2010	0	adult	M		yes		2	
2010-07	4	2	2010	18-07-2010	0	adult	F		yes		1	
2010-09	9	1	2010	20-07-2010	0	adult	M		yes		2	
2010-10	2	5	2010	21-07-2010	0	subadult	F		yes		2	
2011-01	2	6	2011	19-06-2011	0	subadult	F				0	Observed from nearby
2011-02	8	3	2011	21-06-2011	0	adult	M		yes		3	
2011-04	10	1	2011	25-06-2011	0	adult	M		yes		3	
2011-05	8	4	2011	26-06-2011	0	adult	M		yes		3	
2011-06	8	5	2011	26-06-2011	0	adult	M				1	
2011-11	2	7	2011	04-07-2011	0	subadult	F		yes		0	Tracing for a couple of days; observed at second occasion
2011-14	2	8	2011	07-07-2011	0	subadult	F		yes		2	
2011-15	8	6	2011	12-07-2011	0	adult	M		yes		3	
2011-16	11	1	2011	20-07-2011	0	adult	F		no		2	
2011-17	2	9	2011	20-07-2011	0	subadult	F		yes		2	
2012-01	10	2	2012	31-05-2012	0	adult	M		yes		2	
2012-02	8	7	2012	09-06-2012	0	adult	M		yes		3	
2012-03	2	10	2012	18-06-2012	0	adult	F	Collar	yes		3	
2012-04	7	2	2012	18-06-2012	0	adult	M		no		3	
2012-05	8	8	2012	19-06-2012	0	adult	M		yes		3	
2012-06	7	3	2012	25-06-2012	0	adult	M		no		3	
2012-07	7	4	2012	27-06-2012	0	adult	M		no		3	
2012-09	3	3	2012	18-07-2012	0	adult	F		yes		3	
2012-12	12	1	2012	20-07-2012	0	adult	M		no		2	
2013-01	10	3	2013	15-06-2013	0	adult	M	#129	yes	N23672 based on painted code on back	3	
2013-04	10	4	2013	20-06-2013	0	adult	M	#129			0	Read code on back
2013-05	3	4	2013	24-06-2013	2	adult	F		yes		3	
2013-07	2	11	2013	25-06-2013	0	adult	F	Collar		N23980 based on GPS positions	0	Distinguished by collar
2013-09	2	12	2013	29-06-2013	0	adult	F	Collar	yes	N23980 confirmed by GPS positions	3	
2013-10	2	13	2013	01-07-2013	0	adult	F	Collar		N23980 based on GPS positions	0	Distinguished by collar
2013-11	2	14	2013	05-07-2013	0	adult	F	Collar		N23980 based on DNA in hairs	3	
2013-12	13	1	2013	11-07-2013	0	adult	F				1	Could have been spotted as a female in a previous year
2013-14	14	1	2013	13-07-2013	0	adult	M		no		3	
2013-15	12	2	2013	16-07-2013	0	adult	M		no	N23495 based on DNA in hairs	1	
2014-01	2	15	2014	11-06-2014	2	adult	F	#53	yes	N23980 based on painted code on back	3	
2014-03	7	5	2014	17-06-2014	0	adult	M		yes		3	
2014-05	2	16	2014	19-06-2014	2	adult	F	Collar	yes	N23980 confirmed by GPS positions	0	Distinguished by collar
2014-06	12	3	2014	28-06-2014	0	adult	M	#57	yes	N23495 based on painted code on back	3	
2014-08	3	5	2014	02-07-2014	2	adult	F		yes		2	
2015-01	2	17	2015	20-06-2015	2	adult	F	Collar	yes	N23980 confirmed by GPS positions	3	
2015-02	2	18	2015	22-06-2015	2	adult	F	Collar	yes	N23980 confirmed by GPS positions	3	
2015-03	7	6	2015	23-06-2015	0	adult	M		yes		3	
2015-04	15	1	2015	24-06-2015	0	subadult	M				1	Could have been spotted as a male subadult in a previous year
2015-06	16	1	2015	25-06-2015	0	subadult	F	#23	yes	N26236 based on painted code on back	3	

Code record	indivID	Rank by individual	Year	Date of arrival	Number of cubs	Age class	Sex	Tags	Ear marks	NPI code	Quality photographs	Comments
2015-08	7	7	2015	29-06-2015	0	adult	M		yes		1	
2015-09	16	2	2015	29-06-2015	0	subadult	F	#23	yes	N26236 based on painted code on back	3	
2015-10	7	8	2015	01-07-2015	0	adult	M		yes		2	
2015-11	17	1	2015	03-07-2015	0	adult	M		no		3	
2015-12	16	3	2015	09-07-2015	0	subadult	F	#23	yes	N26236 based on painted code on back	2	
2015-13	2	19	2015	26-07-2015	2	adult	F	Collar	yes		3	
2015-14	7	9	2015	26-07-2015	0	adult	M		yes		3	
2015-15	7	10	2015	27-07-2015	0	adult	M		yes		3	
2016-01	18	1	2016	29-05-2016	0	subadult	F		no		2	
2016-02	16	4	2016	05-06-2016	0	subadult	F		yes		3	
2016-03	18	2	2016	05-06-2016	0	subadult	F		yes		1	
2016-04	18	3	2016	06-06-2016	0	subadult	F		no		3	
2016-05	7	11	2016	06-06-2016	0	adult	M		yes		3	
2016-06	7	12	2016	07-06-2016	0	adult	M		yes		3	
2016-07	19	1	2016	08-06-2016	0	subadult	M		yes		3	
2016-08	16	5	2016	12-06-2016	0	subadult	F				0	Traced from distance
2016-09	16	6	2016	15-06-2016	0	subadult	F		yes		3	
2016-10	8	9	2016	27-06-2016	0	adult	M		yes		3	
2016-11	2	20	2016	04-07-2016	0	adult	F	Collar		N23980 confirmed by GPS positions	0	Distinguished by collar
2016-12	8	10	2016	06-07-2016	0	adult	M		yes		2	
2016-13	2	21	2016	08-07-2016	0	adult	F	Collar	yes	N23980 confirmed by GPS positions	0	Distinguished by collar
2016-14	2	22	2016	12-07-2016	0	adult	F	Collar	yes	N23980 confirmed by GPS positions	3	
2016-15	8	11	2016	16-07-2016	0	adult	M		yes		1	
2017-01	16	7	2017	11-06-2017	0	adult	F		yes		2	
2017-02	19	2	2017	15-06-2017	0	subadult	M		yes		2	
2017-03	2	23	2017	16-06-2017	2	adult	F	Collar	yes	N23980 confirmed by GPS positions	3	
2017-05	7	13	2017	18-06-2017	0	adult	M		yes		3	
2017-06	2	24	2017	19-06-2017	2	adult	F	Collar	yes	N23980 confirmed by GPS positions	2	
2017-07	19	3	2017	26-06-2017	0	subadult	M		yes		3	
2017-08	2	25	2017	28-06-2017	2	adult	F	Collar	yes	N23980 confirmed by GPS positions	3	
2017-09	2	26	2017	29-06-2017	2	adult	F	Collar	yes	N23980 confirmed by GPS positions	3	
2017-10	20	1	2017	30-06-2017	0	subadult	F		yes		3	
2017-21	21	2	2017	01-07-2017	0	adult	F		yes		2	
2017-11	14	2	2017	04-07-2017	0	adult	M		no		3	
2017-12	3	6	2017	05-07-2017	2	adult	F	Lost collar	yes	Confirmed that N23979 had lost collar	3	
2017-14	16	8	2017	08-07-2017	0	adult	F		yes		2	
2017-15	16	9	2017	16-07-2017	0	adult	F		yes		2	
2017-18	16	10	2017	28-07-2017	0	adult	F		yes		3	
2018-01	22	1	2018	01-06-2018	0	adult	M		yes		3	
2018-02	2	27	2018	03-06-2018	2	adult	F				1	
2018-03	23	1	2018	07-06-2018	0	adult	M		yes		2	Could have been spotted as a male (tagged) in a previous year
2018-05	24	1	2018	20-06-2018	0	subadult	F		no		3	
2018-06	24	2	2018	21-06-2018	0	subadult	F		no		0	Traced from distance
2018-07	16	11	2018	30-06-2018	1	adult	F		yes		3	
2018-08	16	12	2018	01-07-2018	1	adult	F		yes		2	
2018-09	16	13	2018	02-07-2018	1	adult	F		yes		3	
2018-10	2	28	2018	22-07-2018	2	adult	F		yes		0	Distinguished by collar
2019-01	NA	1	2019	26-05-2019	2	adult	F				0	Tracks in snow, female and 2 cubs. Could be a previous female
2019-02	25	1	2019	02-06-2019	0	subadult	M		yes		2	
2019-03	2	29	2019	09-06-2019	0	adult	F	Collar	yes	N23980 confirmed by GPS positions	0	Distinguished by collar
2019-04	16	14	2019	11-07-2019	0	adult	F		yes		3	
2019-05	16	15	2019	12-07-2019	0	adult	F		yes		1	
2019-06	2	30	2019	22-07-2019	0	adult	F	Collar	yes	N23980 confirmed by GPS positions	0	Distinguished by collar
2020-01	26	1	2020	21-07-2020	0	subadult	F		no		3	
2020-03	27	1	2020	23-07-2020	0	adult	M		no		3	Could have been spotted as a male (untagged) in a previous year
2021-01	26	2	2021	20-7-2021	0	adult	F		no		2	

Code record	indivID	Rank by individual	Year	Date of arrival	Number of cubs	Age class	Sex	Tags	Ear marks	NPI code	Quality photographs	Comments
2021-02	28	1	2021	21-7-2021	0	adult	M		no		1	Could have been spotted as a male (untagged) in a previous year
2021-03	2	31	2021	27-7-2021	2	adult	F	Collar	yes	N23980 confirmed by GPS positions	2	
2022-01	29	1	2022	27-05-2022	0	subadult	F		yes		0	Observed simultaneously with next record
2022-02	30	1	2022	27-05-2022	0	subadult	M	#49	yes	N26391 based on painted code	3	
2022-03	16	16	2022	28-05-2022	0	adult	F	Collar	yes	N26236 confirmed by GPS positions	3	
2022-04	31	1	2022	07-06-2022	0	adult	F		yes		1	Could have been spotted as a female (tagged) in a previous year
2022-05	30	2	2022	08-06-2022	0	subadult	M	#49	yes	N26391 based on painted code	2	
2022-06	32	1	2022	08-06-2022	0	adult	M		no		3	Could have been spotted as a male (untagged) in a previous year
2022-07	30	3	2022	08-06-2022	0	subadult	M	#49	yes	N26391 based on painted code	2	
2022-08	30	4	2022	09-06-2022	0	subadult	M	#49	yes	N26391 based on painted code	2	
2022-09	30	5	2022	10-06-2022	0	subadult	M	#49	yes	N26391 based on painted code	1	
2022-10	33	1	2022	10-06-2022	0	adult	F		no		2	Could have been spotted as a female (untagged) in a previous year
2022-11	30	6	2022	16-06-2022	0	subadult	M	#49	yes	N26391 based on painted code	2	
2022-12	34	1	2022	26-06-2022	0	adult	M		yes		2	Could have been spotted as a male (tagged) in a previous year
2022-14	35	1	2022	26-06-2022	0	adult	M		no		2	Could have been spotted as a male (untagged) in a previous year
2022-15	30	7	2022	03-07-2022	0	subadult	M	#49	yes	N26391 based on painted code	2	
